# Warming and leaf litter functional diversity, not litter quality, drive decomposition in a freshwater ecosystem

**DOI:** 10.1038/s41598-020-77382-7

**Published:** 2020-11-23

**Authors:** Gustavo H. Migliorini, Gustavo Q. Romero

**Affiliations:** 1 Programa de Pós-Graduação em Biologia Animal, Universidade Estadual Paulista “Júlio de Mesquita Filho”, São José do Rio Preto, SP Brazil; 2 Laboratório de Interações Multitróficas e Biodiversidade, Departamento de Biologia Animal, Universidade Estadual de Campinas, Campinas, SP Brazil

**Keywords:** Ecology, Climate-change ecology, Ecosystem ecology, Freshwater ecology, Tropical ecology

## Abstract

Environment, litter composition and decomposer community are known to be the main drivers of litter decomposition in aquatic ecosystems. However, it remains unclear whether litter quality or functional diversity prevails under warming conditions. Using tank bromeliad ecosystems, we evaluated the combined effects of warming, litter quality and litter functional diversity on the decomposition process. We also assessed the contribution of macroinvertebrates and microorganisms in explaining litter decomposition patterns using litter bags made with different mesh sizes. Our results showed that litter decomposition was driven by litter functional diversity and was increasingly higher under warming, in both mesh sizes. Decomposition was explained by increasing litter dissimilarities in C and N. Our results highlight the importance of considering different aspects of litter characteristics (e.g., quality and functional diversity) in order to predict the decomposition process in freshwater ecosystems. Considering the joint effect of warming and litter traits aspects allow a more refined understanding of the underlying mechanisms of climate change and biodiversity shifts effects on ecosystem functioning.

## Introduction

Warming is predicted to profoundly impact biodiversity and ecosystems in all biomes on Earth^1^. Global warming is expected to alter the individual’s metabolic rates^2^, distribution of species and their phenology^3^, the structure of communities^4^, species interactions^5^, and ecosystem functioning^6–9^. Concomitantly, changes in biodiversity are occurring at unprecedented rates^10^. Thus, understanding the effects of warming and biodiversity shifts should be a priority issue since these two threats are predicted to impact communities and ecosystems, simultaneously^11^.

Although plant species richness and identity directly influence ecosystem processes and food web structure^12^, it has been argued that the species functional traits are stronger predictors of ecosystem functioning^13^. Recent studies suggest that the distribution of species functional traits within communities, i.e., the trait composition, plays a key role in driving ecosystem processes^14,15^. Functional differences may lead to a variety of interactions among species, and because of the complexity of these interactions it may be difficult to predict the effects of losses or introductions of species in the communities^16^.

Most of the knowledge on biodiversity-ecosystem function (B-EF) relationships come from experiments focusing on plant productivity, while other fundamental processes such as decomposition are relatively less studied^17^ but see^18,19^, especially in aquatic ecosystems. The decomposition of plant organic matter is one of the most important ecosystem processes in freshwater ecosystems since it regulates the cycling of carbon (C) and nutrients, and the efflux of greenhouse gasses such as carbon dioxide (CO_2_) and methane (CH_4_), which can have positive feedbacks to climate change^20,21^. Dead leaves that fall from trees provide energy and substrate to a wide variety of organisms in freshwater ecosystems^19^. Chemical and physical traits in these dead leaves determine the nutritional value, as well as the lability and toxicity of the litter for invertebrate detritivores and microbial decomposers^19^. The suite of chemical traits defines the litter quality and has been shown to be the major determinant of litter decomposition at different environments and latitudes^22^. While some litter species are nutrient-rich and composed of labile carbon (i.e., high quality litter), which benefit decomposers, others may have high concentrations of some secondary compounds and recalcitrant carbon such as lignin (low quality litter), which may inhibit or hinder the activity of microbial decomposers and invertebrate detritivores^19^. On the other hand, the variation in litter trait values at the community level, i.e., the functional diversity, has been shown to control litter decomposition through synergistic mechanisms (complementarity or facilitation), leading to a more efficient utilization of resources among litter consumers^15,23^, and thus, affecting the process of matter transformation and nutrient cycling^16^. For example, nutrient transfer via fungal hyphae or passive leaching among different litter types may improve the resource availability for consumers leading to accelerated decomposition^24,25^. On the other hand, leaching of inhibitory compounds (e.g., secondary compounds) from some leaf species may slow decomposition of neighbor species (antagonistic effect)^19^. However, few studies contrasted the importance of each litter trait aspect (quality or functional diversity) to drive decomposition in freshwater ecosystems, which might help understanding the dynamics of this function, as the control of this process encompasses complex interactions between abiotic (e.g., climate) and biotic factors (e.g., litter diversity and decomposers)^26–28^. Understanding the mechanisms that cause the effects of litter diversity may help ecologists to predict the impacts of species losses and shifts in plant communities and the consequent effects on freshwater ecosystems. This is particularly relevant in a context of environmental change, in which decrease in plant species diversity, shifts in species composition or even on their characteristics (chemical and physical) can result in changes in quality and functional diversity of litter and, hence, affect communities and ecosystems that depend on this resource^29^.

Decomposition depends strongly on temperature^20^ and it is expected to be sensitive to climate warming since increased temperature accelerate litter mass loss directly by leaching and indirectly by increasing the energy intake and, hence, the litter consumption by invertebrate detritivores and microbial decomposers^30–32^. Besides that, the magnitude of temperature impacts on litter decomposition rates may not be as simple to predict because of other factors, such as microbial activity, invertebrate detritivore density and litter quality^9,22,33^, which can influence how the process will respond to such environmental change. A global experiment suggested that warming should increase microbial decomposition but also decrease decomposition mediated by invertebrate detritivores in streams, which would result in unchanged overall decomposition^33^. However, warming can substantially impact food webs and ecosystems because microbial decomposers and invertebrate detritivores drive decomposition in different ways. Microbial decomposition converts a greater proportion of organic compounds to CO_2_^34,35^, whereas invertebrate detritivores transform coarse particulate litter to fine particulate and dissolved organic matter^36^. Litter quality, as represented by chemical constituents, has been shown to influence how temperature affects decomposition, being low-quality litter more sensitive to temperature compared with high-quality litter. However, this response may depend on the litter species present^37^. Therefore, knowing how temperature interacts with other drivers, such as litter quality and functional diversity, is of crucial importance to predict the consequences of rising temperatures on decomposition.

**Figure 1:**
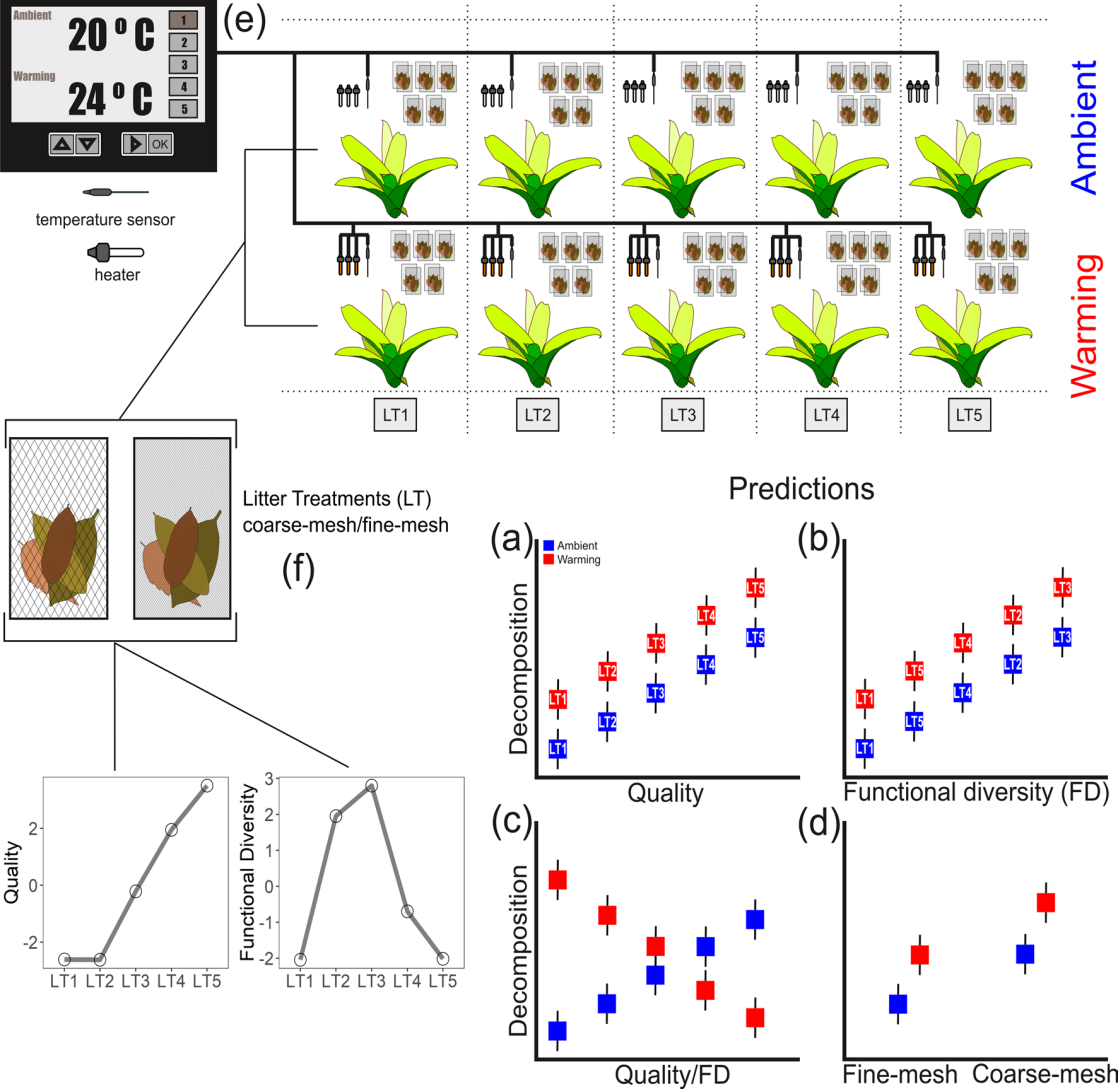
Schematic representation of the experimental design, temperature control system and main predictions. Based on quality hypothesis (**a**), decomposition should increase with litter quality; however, functional diversity hypothesis (**b**) assumes that decomposition increases with higher litter trait dissimilarities. Moreover, warming should exacerbate such relationships by accelerating microbial activity and macroinvertebrate consumption rates. (**c**) Alternatively, warming could affect decomposition rates by interacting with the different levels of quality or functional diversity. (**d**) Decomposition rates are expected to be higher in the coarse-mesh bags and warming may exacerbate the effect; alternatively, warming could decrease litter decomposition in the coarse-mesh bags by negatively affecting macroinvertebrate composition. (**e**) Experimental design: ten tank bromeliads per block (five blocks). Warming effect was achieved by increasing ambient temperature in 4 °C, following the projection for the year 2100. (**f**) Litter treatments (LT) consisted of five mixtures of four out of twelve litter species selected based on their chemical traits, ranging from low to high quality, using coarse and fine-mesh bags. In addition, these litter treatments represented a gradient of functional diversity (see Fig. 2 on how we assessed quality and functional diversity).

Here, we investigated the effects of warming and shifts in litter composition (quality and functional diversity) on decomposition in a freshwater ecosystem. We conducted an experiment using natural microcosms (tank bromeliads) to manipulate litter mixtures differing in quality and functional diversity and water temperature according to projections of future global warming^38^. We also manipulated invertebrate access to the litter using coarse and fine-mesh bags to contrast their contribution with those by microbial decomposers. Natural freshwater microcosms, such as tank bromeliads, are excellent model systems to investigate the influence of climate change on community structure and ecosystem functioning^39–42^ because they are real ecosystems with complex food webs, but the small size allows high replicability and manipulation, besides being susceptible to natural environmental variance^43^. We evaluated the importance of quality and functional diversity of litter, and how they interact with increased temperature to affect decomposition in the presence and absence of macroinvertebrates. We expected that both quality and functional diversity would control decomposition. Decomposition should increase with litter quality as it provides more nutritive resources for microbial decomposers and invertebrate detritivores^19^ (Fig. 1a). On the other hand, decomposition would increase in mixtures with high litter functional diversity, as increasing trait dissimilarity provides complementary resources for microbial decomposers and invertebrate detritivores^15,16,23^, but see^44^ (Fig. 1b). Also, warming would accelerate decomposition rates through its effects on metabolic rates of microbial decomposers and invertebrate detritivores^2^, exacerbating the effects of quality and functional diversity (Fig. 1a,b). Alternatively, warming could accelerate decomposition of low-quality litter since such litter types may be more sensitive to temperature than high-quality litter^37^; we predict that this phenomenon would also occur for functional diversity (Fig. 1c). In addition, decomposition rates would be higher in the presence of macroinvertebrates, which would be boosted by warming and increasing litter quality or functional diversity (Fig. 1d). Alternatively, warming could decrease decomposition in the coarse-mesh bags by negatively affecting macroinvertebrate community composition^4,45^.

## Material and methods

### Study area and organisms

We conducted our study in a closed *restinga* (i.e., coastal sand-based) forest within Parque Estadual Serra do Mar—Núcleo Picinguaba (23°21′27″ S, 44°51′01″ W), an Atlantic forest conservation area situated in the north coast of São Paulo state, Brazil. The *restinga* forest is characterized by the presence of trees approximately 15 m tall with many epiphytic plants, mainly bromeliads. *Neoregelia johannis* (Carriére) L. B. Smith. (Bromeliaceae) is one of the most abundant bromeliad species in the area with leaves that can be over 1 m long and hold more than 2 L of water in the tanks formed at their base^46^. Phytotelm bromeliads can house a wide diversity of arthropod fauna, mainly insects in their larval stages, including predators (Tabanidae, Tanypodinae, Zygoptera, Dytiscidae, Corethrellidae), detritivores (Trichoptera, Limoniidae, Scirtidae, Syrphidae, Psychodidae, Chironomidae) and filter feeders (Culicidae). In addition, a diverse terrestrial fauna inhabits the non-submerged parts of bromeliad leaves, including spiders, mites, harvestmen and collembolas^47,48^.

### Leaf litter collection and chemical analyses

We chose 12 native tree species from closed *restinga* that are abundant at the field site: *Jacaranda puberula* (Cham.), *Inga subnuda* (Salzm.), *Alchornea triplinervia* (Spreng.), *Pera glabrata* (Schott), *Myrcia glabra* (O. Berg), *Myrcia racemosa* (O. Berg), *Andira anthelmia* (Vell.), *Abarema brachystachia* (DC.), *Cupania oblongifolia* (Mart.), *Miconia* sp., *Lacistema pubescens* (Mart.), *Inga edulis* (Mart.)*.* We cut branches of each species in October 2014 and air-dried them at room temperature until the leaves fall and we used them for the chemical analyses and the experiment. Carbon (C) and nitrogen (N) concentrations were quantified by dry combustion in an elemental analyzer (Perkin Elmer 2400 II CHN) and phosphorus (P) concentration was determined through colorimetry by the vanadate-molybdate method^49^. We used these data to calculate the carbon to nitrogen ratio (C:N) and nitrogen to phosphorus ratio (N:P) for each litter species (Supplementary Table S1). Also, we quantified lignin concentration with the acid-detergent method^50^, and litter tannins and total phenolics concentrations using the Folin-Ciocalteu method^51^. C and N analyses were performed at the Analytical Center of the Institute of Chemistry—University of Campinas. Lignin, P, tannins and total phenolics were analyzed at the Center of Nuclear Energy in Agriculture (CENA)—University of São Paulo.

### Leaf litter treatments

**Figure 2:**
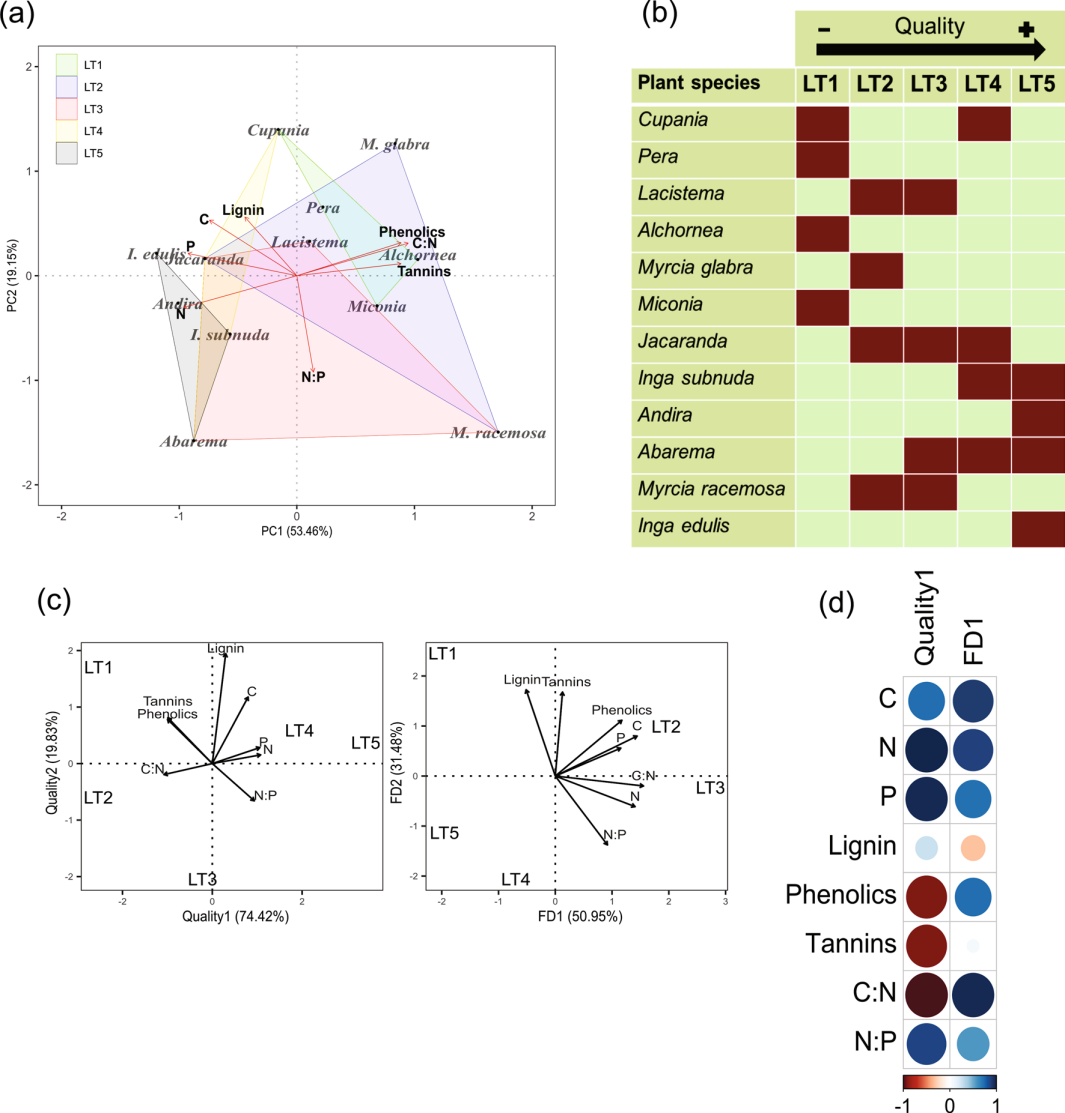
Analytical procedure used to set the litter treatments. (**a**) We used a principal component analysis to summarize the trait matrix of our 12 leaf litter species. (**b**) Then we selected five litter treatments (LT) with four litter species according to their proximity with traits in the multivariate space of the PCA bi-plot, ranging from low to high quality. (**c**) We conducted a PCA on the mean values (CWM) of each trait in each LT and retained the first component as predictor of litter quality. We also conducted a PCA on Rao’s Q values from each trait across the five LTs and retained the first principal component (FD1) as a predictor of the effect of functional diversity. (**d**) Quality1 was positively loaded by concentrations of N and P, and negatively loaded by phenolics, tannins and C:N ratios. FD1 was positively correlated with dissimilarities of C, N and C:N ratios.

To quantify and visualize the trait differences among our 12 litter species, we conducted a principal components analysis (PCA; *PCA* function, FactoMineR package v. 2.3) using all chemical traits listed above, previously standardized with *z*-scores (Fig. 2a, Supplementary Table S1), which allowed us to visually determine five litter treatments (hereafter LT; each one containing four litter species) based on the position of each species (i.e., proximity with traits) in the litter trait space. The LTs represent a gradient of quality, ranging from low to high, which determines the expected rate at which they should decompose (Fig. 2a,b). Low quality LT was composed by litter having high concentrations of structural and secondary compounds but low concentrations of nutrients (LT1), whereas high quality litter was composed by nutrient-rich litter with lower concentrations of structural and secondary compounds (LT5), with intermediate quality litter distributed between these extremes (LT2–LT4). The LT1 included *C. oblongifolia*, *P. glabrata*, *A. triplinervia*, and *Miconia* sp.; LT2 included *L. pubescens*, *M. glabra*, *J. puberula*, and *M. racemosa*. The LT3 was composed by *L. pubescens*, *J. puberula*, *A. brachystachya*, and *M. racemosa*. LT4 contained *C. oblongifolia*, *J. puberula*, *I. subnuda*, and *A. brachystachya*. Finally, the LT5 included *I. subnuda*, *A. anthelmia*, *A. brachystachya,* and *I. edulis* (Fig. 2b).

To assess the role of litter quality, we calculated the community-weighted mean values (CWM) of each trait in each LT assuming that greater trait values would drive the effects of our litter clusters on decomposition^52^. CWM is often used to describe the functional composition of communities which varies according to the abundances of species^53^. Since the abundance of each species was evenly distributed in our LTs, differences in the CWM values were directed to their trait values. CWM values were calculated using the *functcomp* function implemented in the FD R-package. After obtaining the mean values of each trait in each treatment, we standardized the values using *z*-scores. Then we conducted a PCA using our mean trait matrix (Fig. 2c). Litter quality was then assessed by retaining the first PCA axis, which explained most of the variance of the mean trait values. The first axis of quality PCA (quality1) was positively loaded by higher concentrations of N and P and negatively loaded by polyphenolics, tannins, and C:N ratio (Fig. 2d).

To determine the importance of litter functional diversity on decomposition, we calculated the Rao’s quadratic entropy (Rao’s Q) for each trait in each LT, using the *dbFD* function implemented in FD package (v. 1.0-12). Then, we conducted a PCA using the Rao’s Q values previously standardized with *z*-scores and retained the first axis (Fig. 2c). Although Rao’s Q is generally used in multi-trait approaches to access trait variability, single-trait Rao’s Q have been used to identify groups of traits driving the variance in the trait matrix^54^. This measure can be used to assess the importance of niche complementarity and assumes that trait differences between litter species drives decomposition through complementary consumption of the different litter species^15^. FD1 axis of litter functional diversity PCA separated our litter treatments mainly by high dissimilarities in C and N concentrations, and in C:N ratios (Fig. 2d). A correlation plot for CWM and Rao values is provided in Supplementary Fig. S1.

### Experimental design

To investigate the mechanisms controlling litter decomposition and the effect of warming on this ecosystem function, we used 50 tank-bromeliads *Neoregelia johannis* acquired from a greenhouse to ensure all plants had similar sizes and water storage capacities and were virtually free of colonizing organisms. Before starting the experiment, we washed the bromeliads with spring water, a 500 mL solution of antibiotic (Ciprofloxacin; 5 µg/mL), and 5% sodium hypochlorite solution to remove invertebrates and unnatural bacteria^39–41^. We planted the bromeliads in the ground of the *restinga* following a randomized block design (5 blocks) and installed plastic roofs at 1 m over each plant to prevent any input of rain and organic matter like branches and fallen leaves from trees but allowing the entrance of colonizing organisms such as microorganisms and insects. Colonization of bromeliads occurred naturally during the experiment as we wanted this to be shaped by our litter and temperature treatments. To keep the water level constant, we watered the bromeliads with stored rainwater every other day.

The warming effect was simulated with an electronic heating system (Fig. 1e) composed by pre-programmed controllers connected to a Delta human–machine interface (HMI). The heating equipment was composed of five boxes (containing the electronic components) connected. Each component box (block) was responsible for accessing and controlling the temperature of 10 bromeliads (five with ambient temperature and five warmed; Fig. 1e). We used submersible water heaters (1 W; n = 3) in half of the bromeliads of each block to maintain a continuous 4 °C increase above the ambient temperature of unheated bromeliads (Supplementary Fig. S2). This increase in temperature was achieved by submersible sensors in the unheated, reference bromeliads, which switched on and off the heaters from treatment bromeliads through the controllers. To account for the physical effect of the heaters we used heaters turned off in the unheated bromeliads. The temperature difference used in this experiment followed the projections of temperature increase in the southeastern region of Brazil over the next century^38,55^. We conducted our experiment from April to July 2015.

For each LT, we placed 0.4 ± 0.05 g of air-dried leaves (0.1 ± 0.01 g of each species; mean ± SD) in coarse (3 mm plus six additional 10 mm openings) and fine-mesh litterbags (0.05 mm), (Fig. 1f). Litter in fine-mesh bags was accessible only by microorganisms, whereas coarse-mesh bags also allowed access of macroinvertebrates, like detritivorous insects. We prepared a total of 250 coarse-mesh and 250 fine-mesh litterbags. In each block, we placed one LT per bromeliad, repeating the treatment in warmed and ambient bromeliads. Each bromeliad received five coarse-mesh and five fine-mesh litter bags in five different wells, being one litter bag of each mesh size per well. One pair of litter bags was retrieved from each bromeliad (50 coarse and 50 fine) after 16, 32, 48, 64 and 80 days. In summary, our experiment had 5 blocks × 2 temperatures × 1 bromeliad × 2 mesh sizes × 5 litterbags, totalizing 100 replicates per each of 5 LT. Litter from each bag was taken out and oven-dried at 60 °C and weighed. Before oven-drying, the coarse-mesh litter was carefully inspected and cleaned under water to remove possible insects which were collected and stored in 80% ethanol solution, quantified, and identified using taxonomic keys^56^ and photos of previous collected specimens (Supplementary Table S2).

### Statistical analyses

All data analyzes were carried out using R version 3.2.2^57^. We measured decomposition using the decay coefficient (*k*) assuming the exponential decay model *m*_*f*_/*m*_*i*_ = e^−*k*t^ where *m*_*f*_ and *m*_*i*_ are the final and initial litter dry mass, respectively, *t* is time in days and *k* is the decomposition coefficient. The decomposition coefficient (*k*) was log-transformed after verifying the parametric assumptions of linear models using plots of residuals for normality and homoscedasticity. In order to verify the differences in the decomposition rates among our LTs, we used a linear mixed model (*lme* function, nlme package v. 3.1-144) with LTs, temperature, mesh size and their interactions. LTs and mesh type were included as fixed predictors and temperature as a continuous variable. Block was included as a random factor. We conducted a pairwise comparison test with Tukey-adjusted *p*-value using the *lsmeans* function of the lsmeans package (v. 2.30) to check the differences between the LTs. We conducted additional models to test the effects of warming, quality, and functional diversity of litter on decomposition using the quality1 and FD1 axes as continuous predictor variables, temperature as a continuous variable, and the interactions between quality1 and temperature, and FD1 and temperature. We conducted separate models for fine-mesh decomposition (microorganisms) and coarse-mesh decomposition (macroinvertebrates + microorganisms) since we included the macroinvertebrates collected inside the coarse-mesh bags as covariates in the coarse-mesh decomposition models.

### Ethics declaration

Samples were collected under permit by SISBIO #45548.

## Results

**Figure 3:**
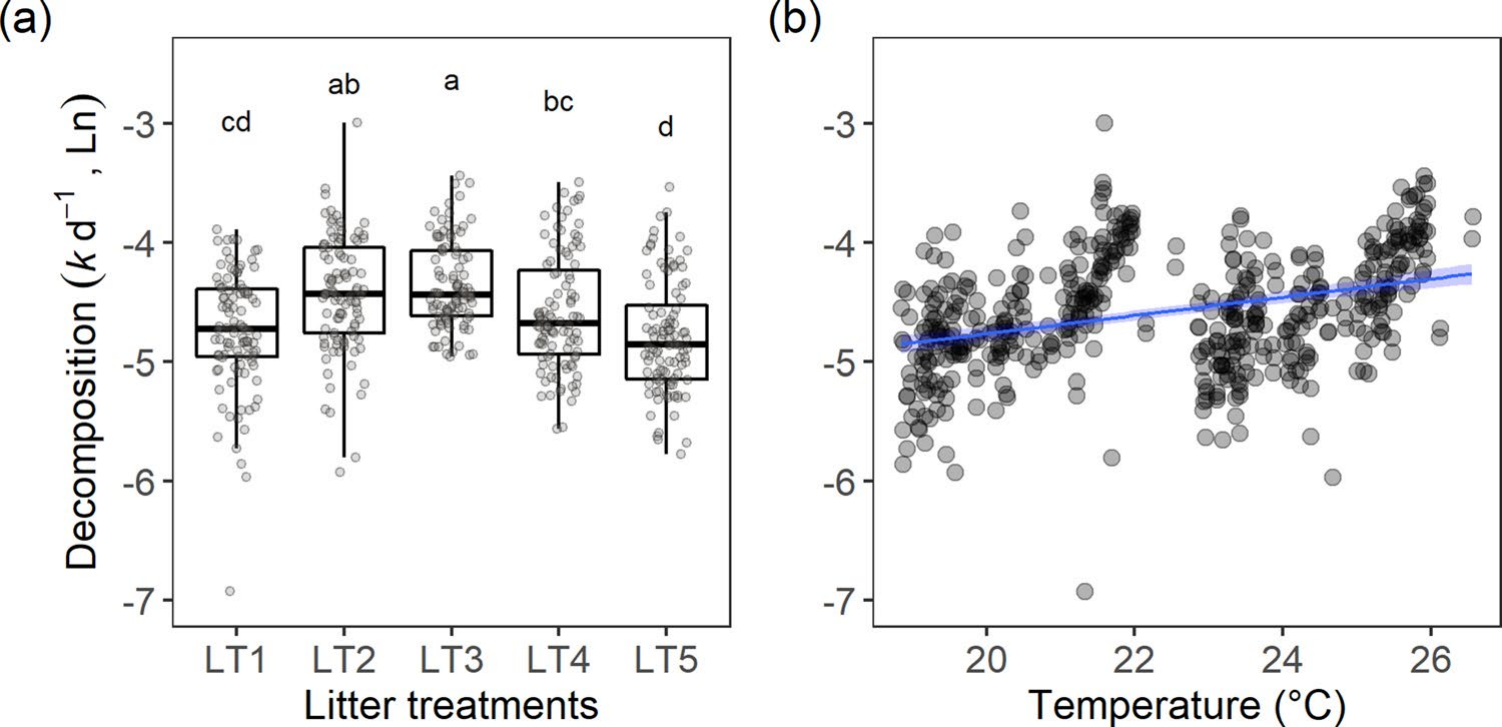
Leaf litter decomposition rates differed among litter treatments (**a**) (N = 100) and increased with temperature (**b**) (r = 0.35, *p* < 0.0001, N = 500). Different letters in (**a**) indicate statistical difference in *p* < 0.05. See definition of LTs in Fig. 1. Points represent data from both fine- and coarse mesh bags.

After 80 days, 56.3% ± 4.6 (LT1), 54.8% ± 3.0 (LT2), 42.2% ± 2.4 (LT3), 60.2% ± 2.4 (LT4) and 66.1% ± 1.9 (LT5) of litter mass were remaining in the fine-mesh bags, and 54.9% ± 4.9 (LT1), 44.4% ± 5.7 (LT2), 47.1% ± 2.4 (LT3), 59.1 ± 3.3 (LT4) and 64.4 ± 3.3 (LT5) of litter mass were remaining in the coarse-mesh bags (mean ± SE). The decomposition rates differed among LTs (Table 1; Fig. 3a) and were positively correlated with temperature (r = 0.35, *p* < 0.0001; Table 1; Fig. 3b). However, decomposition rates did not differ between mesh sizes (Table 1; Supplementary Fig. S3), meaning that microorganisms, and not macroinvertebrate detritivores, controlled the detrital processing. No significant interaction effect was detected for LTs, temperature and mesh size. The pairwise test revealed that the decomposition rates differed between LT1 and LT2 (t = − 4.568, *p* = 0.0001), LT1 and LT3 (t = − 5.665, *p* < 0.0001), LT2 and LT5 (t = 5.857, *p*  < 0.0001), LT3 and LT4 (t = 3.397, *p*  = 0.0066), LT3 and LT5 (t = 6.957, *p*  < 0.0001), LT4 and LT5 (t = 3.564, *p*  = 0.0037) (Fig. 3a).

**Table 1:** Results of linear mixed effect models examining how decomposition rates changed in response to litter treatments (LT), temperature, mesh size (fine *versus* coarse) and interactions.

Source	numDF	denDF	F-value	*p* value
(intercept)	1	476	24,219.686	< 0.0001
LT	4	476	18.694	< 0.0001
Temperature	1	476	74.329	< 0.0001
Mesh	1	476	0.121	0.729
LT × Temperature	4	476	0.433	0.785
LT × Mesh	4	476	1.338	0.255
Temperature × Mesh	1	476	1.140	0.286
LT × Temp. × Mesh	4	476	0.366	0.833

Marginal R^2^ = 0.24, conditional R^2^ = 0.25. Significance at *p* < 0.05. numDF = numerator degrees of freedom, denDF = denominator degrees of freedom.

**Figure 4:**
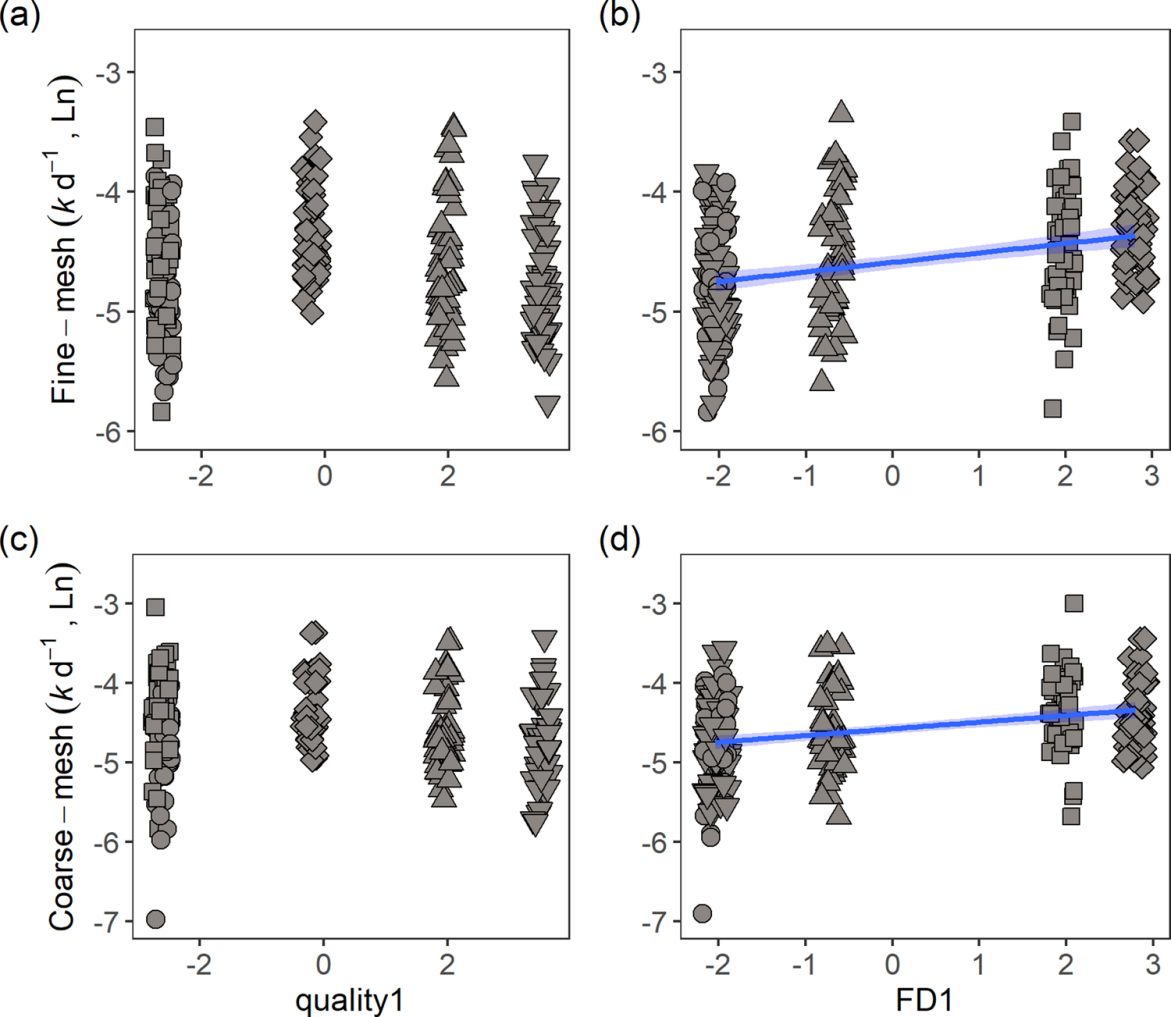
Decomposition rates did not increase with increasing litter quality but increased with increasing litter functional diversity in fine-mesh bags (**a**–**b**; r = 0.34, *p* < 0.0001) and coarse-mesh bags (**c**–**d**; r = 0.33, *p* < 0.0001). Data represent litter quality and functional diversity gradient of litter treatments (LT). Circle: LT1, square: LT2, rhombus: LT3, triangle: LT4, inverted triangle: LT5. See definition of LTs in Fig. 1. Quality1 and FD1 are the first axes of PCA on litter mean trait values and RaO’s Q values, respectively.

In both fine and coarse-mesh bags, litter functional diversity, not litter quality, accelerated the decomposition rates (Table 2; Fig. 4). Moreover, decomposition increased with increasing temperature (fine-mesh: r = 0.40, *p*  < 0.0001, coarse-mesh: r = 0.30, *p*  < 0.0001; Table 2). The positive effect of litter functional diversity indicated higher decomposition with increasing dissimilarities in C (fine-mesh: F_1,241_ = 25.18, *p*  < 0.0001; coarse-mesh: F_1,241_ = 29.72, *p*  < 0.0001) and N (fine-mesh: F_1,241_ = 18.83, *p*  < 0.0001; coarse-mesh: F_1,241_ = 3.89, *p*  = 0.0497) concentrations (Supplementary Fig. S4a–d). No interaction effect was detected in the models for both mesh sizes. Additionally, the decomposition rates in the coarse-mesh bags were not affected by richness and abundance of detritivorous insects collected inside the litter bags. A comparison between models with FD1 and models with dissimilarities of C and N as predictor variables revealed that for microbial decomposition (fine-mesh), the model with litter dissimilarities best explained decomposition (AIC = 273.5 and 268.8, respectively). For coarse-mesh, the model including trait dissimilarities showed higher AIC (332.8) compared to the model with FD1 as a predictor variable (AIC = 329.7), which may suggest trait dissimilarities besides C and N to be important for decomposition mediated by microorganisms and invertebrates.

**Table 2:** Results of linear mixed effect models examining how decomposition rates changed in response to litter quality, litter functional diversity (FD) and temperature in (a) fine-mesh bags and (b) coarse-mesh bags.

Source	Estimate	Std. Error	DF	t-value	*p* value
*(a) Fine-mesh (microbial)*					
(Intercept)	− 6.441	0.262	242	− 24.590	< 0.0001
Quality1	0.001	0.011	242	0.105	0.917
FD1	0.076	0.014	242	5.492	< 0.0001
Temperature	0.083	0.012	242	7.104	< 0.0001
*(b) Coarse-mesh (total)*					
(Intercept)	− 6.011	0.300	240	− 20.013	< 0.0001
Quality1	− 0.012	0.013	240	− 0.913	0.362
FD1	0.077	0.016	240	4.973	< 0.0001
Temperature	0.065	0.013	240	5.024	< 0.0001
Detritivores richness	− 0.003	0.023	240	− 0.134	0.894
Detritivores abundance	− 0.001	0.004	240	− 0.368	0.713

Interaction between Quality1 and Temperature, and FD1 and Temperature were not significant, thus were removed to increase model robustness. Quality1 and FD1 are the first PCA axes on the mean trait values and Rao’s Q values, respectively. Richness and abundance of detritivores collected inside the coarse-mesh bags were included in the coarse-mesh model as covariates. Fine-mesh marginal R^2^ = 0.26, conditional R^2^ = 0.26. Coarse-mesh marginal R^2^ = 0.19, conditional R^2^ = 0.21. Significance at *p* < 0.05.

## Discussion

Although the effects of litter diversity and their traits on decomposition are well documented in both terrestrial and aquatic ecosystems^28^, the mechanisms underlying litter effects are still poorly understood (but see García-Palacios et al.^54^), especially in tropical regions. Likewise, little is known on how decomposition will respond to climate changes along with shifts in plant composition^42,58^. As far as we know, this is the first decomposition study that investigated the combined effects of predicted global warming and litter composition, by partitioning litter effects into quality and functional diversity. Here we show that litter functional diversity may be the main driver of decomposition in tropical freshwater ecosystems, increasing complementarity potentially via complementary resource use and nutrient transfer among litter species^25^. Our results indicate that litter decomposition may be more dependent on how different leaf species are combined in terms of traits rather than on the concentration of nutrients or secondary and structural compounds. Additionally, our findings suggest that increasing the homogenization of trees species by loss or changes in the composition may have substantial impacts on the ecosystem functioning of aquatic environments. Moreover, warming independently of the litter functional diversity accelerated the rates of litter breakdown in our study, which was mainly microbial driven.

Litter decomposition in freshwater systems provides a substantial amount of CO_2_ to the atmosphere, contributing significantly to the global C cycle^59^. Therefore, it is important to understand how climatic changes will affect the processing of organic matter in these systems. Our study demonstrated that microorganisms play a key role in litter decomposition and increasing temperature by 4 °C as predicted for the end of this century may accelerate microbial decomposition in tropical aquatic systems. This is particularly important because decomposition in tropical freshwater ecosystems is mostly driven by microorganisms (i.e., bacteria and fungi)^33^. Although such findings have emerged from studies in streams, they are not limited to these ecosystems. Previous studies using tank bromeliads reported similar patterns in litter decomposition^41,48,60^. Indeed, in our study we did not detect differences in decomposition between the two mesh sizes after 80 days, which reinforces the role of microorganisms as the main decomposers in tank bromeliads. However, we cannot rule out the possibility that due to our design (i.e., fine-mesh and coarse-mesh were in the same tank compartment), fauna effect on litter in coarse-mesh bags may have boosted microbial communities inside fine-mesh bags via microbial and nutrients exchange**.** In addition, one could argue that the lack of time for colonization by insects before starting the experiment could have influenced the results. However, colonization was fast as it can be observed in Supplementary Table S2. Besides that, in a recent study the authors manipulated tank bromeliads to have only bacteria or to be colonized by bacteria and macroinvertebrates and found no difference in decomposition rates^41^, supporting our findings here.

Interestingly, litter functional diversity arose as the main driver of litter decomposition, regardless of the presence or absence of invertebrate detritivores. Litter functional diversity hypothesis predicts that higher trait dissimilarity increases decomposition through resource complementarity for microbial decomposers and invertebrate detritivores^15,54,61,62^. In our study, decomposition was enhanced by increasing dissimilarity in carbon and nitrogen, in both microbial (fine-mesh) and total (coarse-mesh) decomposition, which is supported by recent studies^15,54^, but see^44^. Contrary to our findings, Frainer et al.^44^ found no evidence for effects of litter functional dissimilarity on decomposition and conclude that litter diversity effects are less pronounced in streams. However, they point out this result as being potentially caused by a lack of statistical power to detect dissimilarity effects in their two-species mixtures. In addition, they point to the possibility of effect suppression in the scale at which their study and analyzes were made, that is, the litter bag scale. Among the mechanisms of litter functional diversity effects on decomposition, nitrogen transfer between litter species has been pointed out as the main mechanism in several studies in terrestrial systems^19,25,54^. Nitrogen transfer among litter species, via passive leaching or active microbial transfer (fungal hyphae), is related to niche complementarity effects, where a nutrient-rich litter enhances the nutritional values of a nutrient-poor litter, increasing the resource availability for microbial decomposers and invertebrate detritivores, and hence increasing the overall decomposition^18,25^. Handa et al.^25^ suggest that nitrogen transfer from N-fixing litter boosted the decomposition of nutrient-poor litter in a global scale experiment in terrestrial and aquatic systems. Indeed, in our study the litter treatment that showed the highest dissimilarities in C and N (LT3), and that tended to decompose at higher rates, contained the N-fixing species *Abarema brachystachya*. We suggest that N transfer from *A. brachystachya* to nutrient-poor species, by leaching or fungal hyphae, may have increased the overall decomposition in these treatments. It is important to mention that decomposition tended to be higher in the intermediate levels of litter quality (LT2 and LT3), which also represented the highest levels of functional diversity. This finding corroborates the results from a recent laboratory study where litter quality was manipulated at different levels and the faster decomposition occurred at the intermediate quality^58^. However, the authors suggest compensatory feeding of detritivores, i.e., enhanced feeding on poor quality food to compensate for the low nutrient intake, as the possible explanation for this result^63,64^. In our study, decomposition was mainly driven by microorganisms, which indicates that compensatory feeding is not the mechanism behind the effect.

Our study identified that litter functional diversity was the main driver of litter decomposition in tank bromeliads. However, it is important to recognize that our design to setup litter treatments did not fully allow disentangling quality from functional diversity. Quality and functional diversity values were both obtained from the same litter treatment, which did not allow testing it in an orthogonal way. Our highest functional diversity treatment was also the intermediate litter quality, and this intermediate quality effect could probably be detected by fitting a non-linear model with polynomial predictor for quality. However, in our predictions we expected the decomposition to increase with our quality gradient in a linear way. Thus, our findings support our predictions, and despite the methodological limitations, can be applied in future studies addressing similar questions and using different study systems.

Our results provide novel evidence on the effects of climate change and litter diversity on litter decomposition in aquatic systems. Warming affected litter decomposition in both mesh sizes, but the rates did not differ between mesh sizes indicating that microorganisms control litter decomposition. Therefore, we suggest that macroinvertebrates may be less sensitive to the predicted warming in tropical regions, and thus will not increase their consumption sufficiently to overcome the microbial effect. Such findings are in accordance with a previous study in tank bromeliads where the authors found that warming did not affect richness and composition of macroinvertebrates^39^. They suggest that these organisms may be resistant or resilient to changes in temperature as they live in small water bodies and are frequently exposed to temperature variation, being adapted to these environment^4^. However, we cannot rule out the possibility that our coarse-mesh bags may have prevented access to litter for some invertebrate detritivores and thus decreased their effect on overall decomposition, despite previous studies in tank bromeliads also did not find effects of invertebrate detritivores on decomposition^41,48,59^. On the other hand, recent studies have shown that although microorganisms may be the major decomposers of organic matter in bromeliads, macroinvertebrates may have an important role in the interactions with microorganisms^41^. In their study, Bernabé et al.^41^ demonstrate that warming may reduce bacterial density by increasing the metabolism of invertebrate detritivores and direct consumption on bacteria biofilm, thus reducing bacterial activity on litter decomposition^65^. It is known that invertebrate detritivores consume the biofilm on litter as it is a nutrient-rich resource^66^. Contrary to our results, Ferreira and Canhoto^67^ found that the invertebrate-driven decomposition was more responsive to the increasing temperature than the microbial decomposition, which accelerated the total decomposition. However, their study was conducted in a temperate region where, unlike in the tropical areas, microorganisms are less active because of colder temperatures^33,68^. Litter decomposition rates are expected to increase with temperature because elevated temperature accelerates biochemical reactions and metabolism of litter consumers^2,33^. Our results highlight the importance of investigating the impacts of climate changes on species interactions to understand the responses of ecosystems.

Contrary to our expectations, we did not find an interactive effect between warming and our gradients of quality and functional diversity. We expected that warming would exacerbate the effects of quality and functional diversity on decomposition by accelerating the consumption rates of microbial decomposers and invertebrate detritivores, as increasing temperature should accelerate metabolic rates of litter consumers^2^. However, our findings suggest that the effects of warming may not depend on litter type (low- or high-quality, low- or high-functional diversity). Such results may be supported by a recent meta-analysis where the authors examined the effects of elevated atmospheric CO_2_ and temperature on litter decomposition in streams^9^. They concluded that the effects of warming on litter decomposition may not depend on litter chemical differences^9^. On the other hand, a global synthesis study on the temperature sensitivity of leaf litter decomposition in streams and rivers analyzed data from 169 studies and found some evidence that the suite of litter chemical traits determines how decomposition is affected by temperature^37^. However, this response was only detected when a particular species (N-fixing species) was included in their analyses^37^. Other manipulative studies should address the interactive effects of temperature and litter trait diversity considering different components of microbial and detritivore communities, in order to predict the functioning of freshwater ecosystems in future scenarios.

Predictive studies combining the joint evaluation of future temperature changes and litter diversity on decomposition are rare. Our study demonstrates that the degree of variation of litter traits, i.e., the functional diversity, rather than litter quality, may be more important to predict litter decomposition. Moreover, future temperature rise may accelerate decomposition rates in tropical aquatic systems, mainly by stimulating the microbial-driven decomposition. Our work reveals the differential impact that warming may have on decomposer communities and highlight the importance of considering different litter trait aspects in order to predict the effects of biodiversity shifts on the functioning of freshwater ecosystems. Future studies should access the different components of microbial communities (e.g., diversity and abundance) to better understand the effects of global warming on decomposition in tropical freshwater ecosystems.

## Supplementary information

Supplementary Information.
