# Supplementary material for: Warming and leaf litter functional diversity, not litter quality, drive decomposition in a freshwater ecosystem
Source: Sci Rep. 2020 Nov 23;10:20333. doi: 10.1038/s41598-020-77382-7 (PMC7684280; doi:10.1038/s41598-020-77382-7)
Supplement: Supplementary file 1 — Supplementary Information. [file 41598_2020_77382_MOESM1_ESM.pdf]

Warming and leaf litter functional diversity, not litter quality, drive decomposition in a freshwater ecosystem

Gustavo H. Migliorini and Gustavo Q. Romero

Supplementary Information

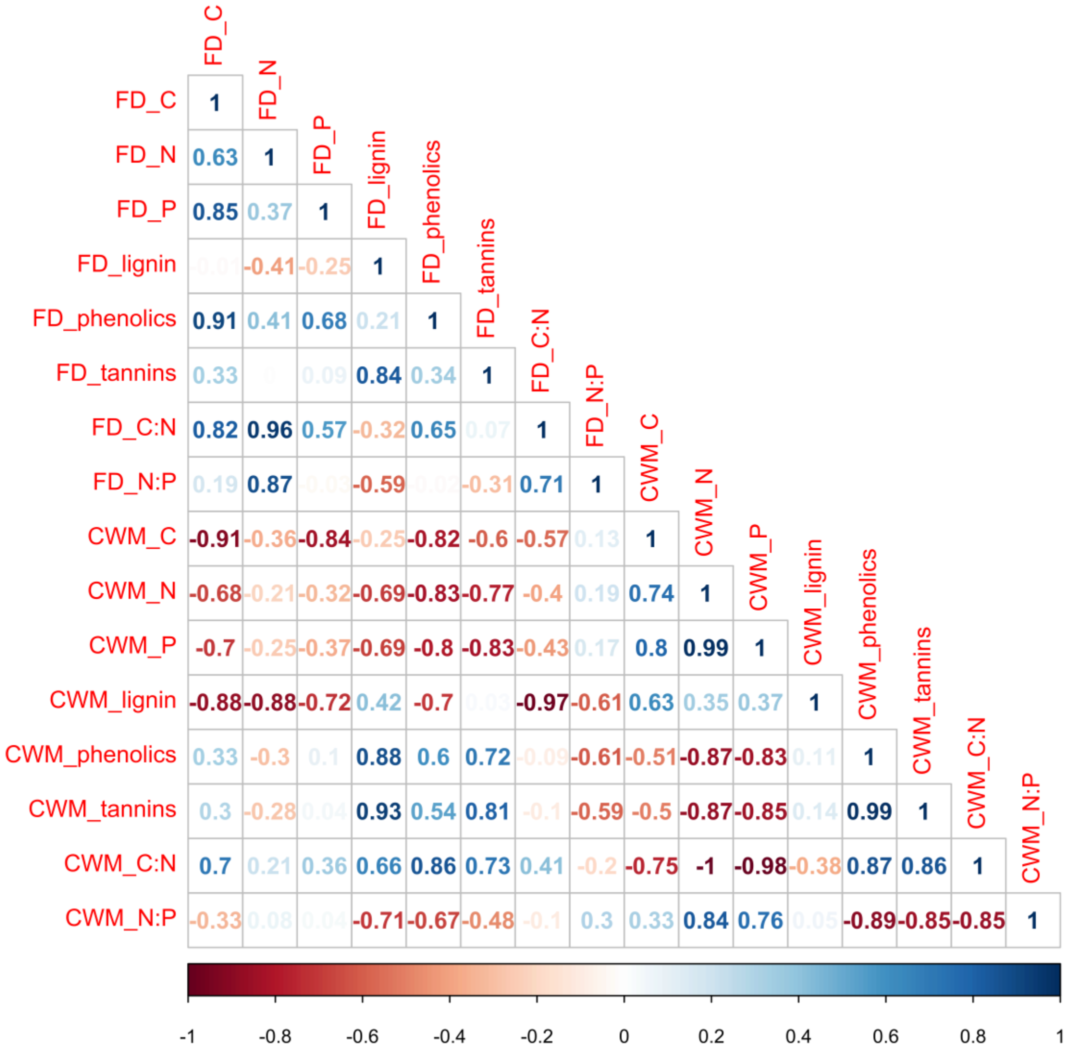

Figure S1. Correlation table between CWM and Rao's Q values of each litter trait measured for the 12 litter species.

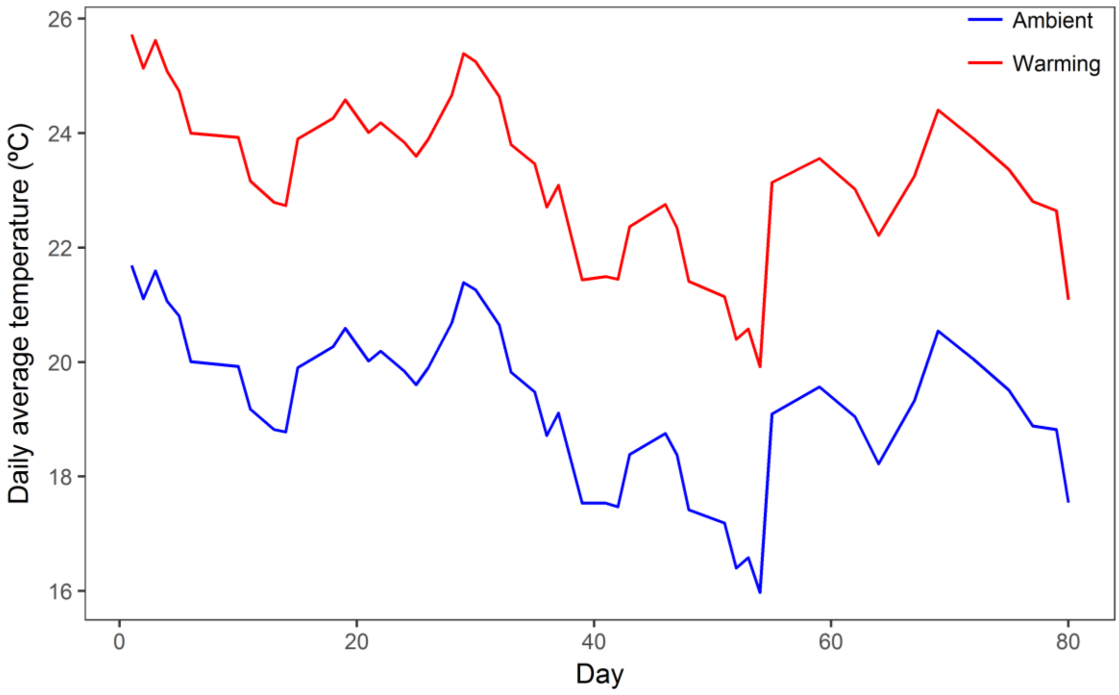

Figure S2. Temperature variation (mean per day) during the study for each temperature scenario.

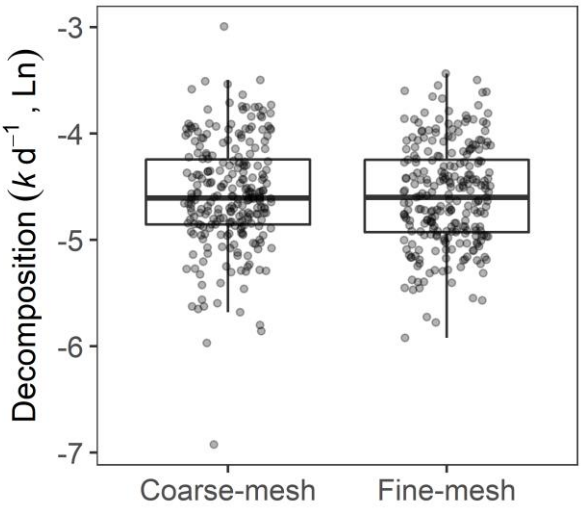

Figure S3. Leaf litter decomposition in coarse-mesh bags (accessed by microorganisms and detritivores) and fine-mesh bags (accessed by microorganisms only).

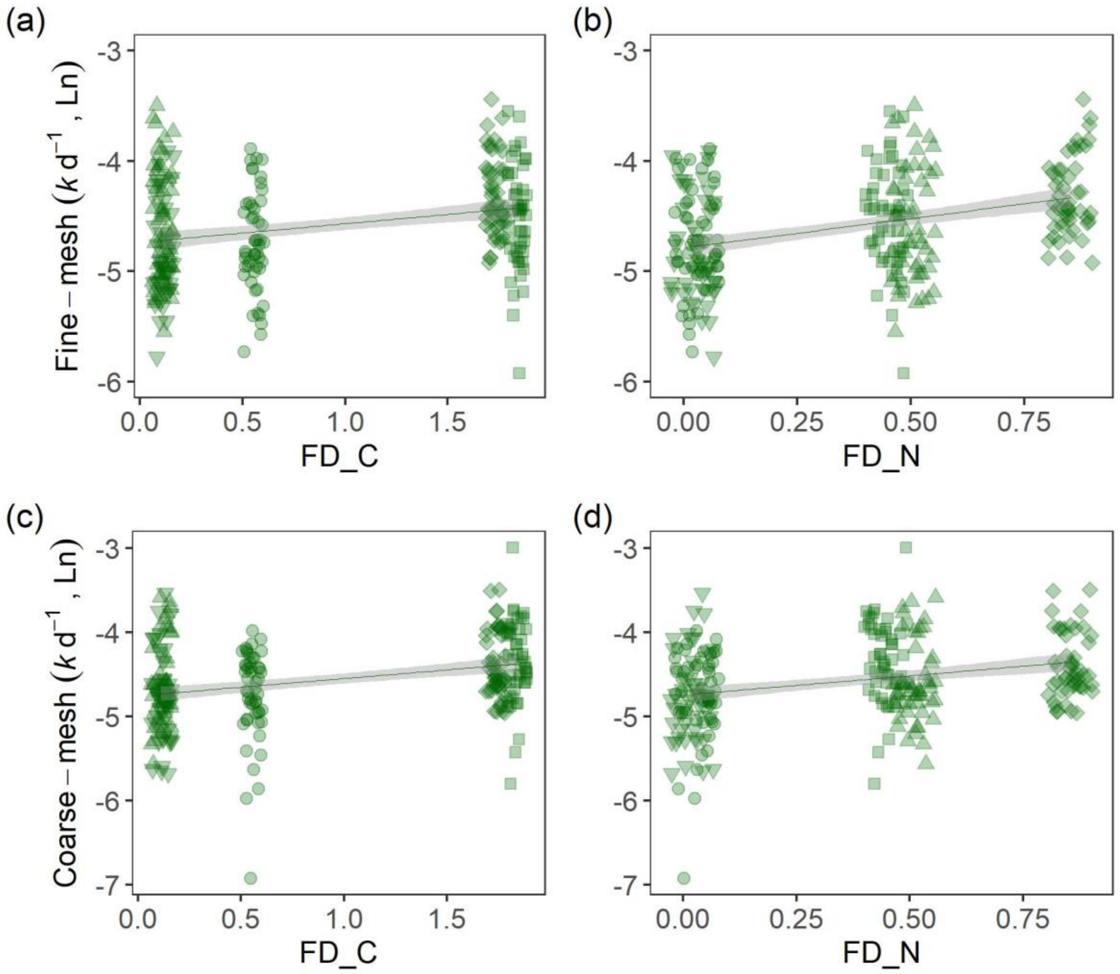

Figure S4. Decomposition rates (*k*) increased with dissimilarities of C and N concentrations, and C:N ratios in (a-c) fine-mesh bags and (d-f) coarse-mesh bags. Data represent litter quality and functional diversity gradient of litter treatments (LT). Circle: LT1, square: LT2, rhombus: LT3, triangle: LT4, inverted triangle: LT5. FD = functional diversity. FD_C: carbon functional dissimilarity, FD_N: nitrogen functional dissimilarity.

Table S1. Leaf litter traits (mean ±SD, n=3) of the 12 species used in the experiment

	C<br>% dry mass	N<br>% dry mass	P<br>g/kg dry mass	Lignin<br>g/kg dry mass	Total phenolics<br>g/kg dry mass	Total tannins<br>g/kg dry mass	C:N ratio	N:P ratio
Jacaranda puberula	48.8 (± 0.93)	2.6 (± 0.38)	1.4 (± 0.30)	201.5 (± 8.10)	41.7 (± 4.27)	34.3 (± 4.50)	18.5(± 2.44)	1.85 (± 0.25)
Inga sbnuda	47.2 (± 0.18)	3.3 (± 0.16)	1.3 (± 0.14)	325.3 (± 2.04)	100.9 (± 5.38)	81.9 (± 4.44)	14.3 (± 0.67)	2.50 (± 0.17)
Alchornea triplinervia	45.6 (± 0.15)	2.1 (± 0.10)	1.0 (± 0.04)	151.2 (± 5.81)	175.9 (± 8.31)	169.1 (± 8.26)	21.9 (± 1.13)	2.02 (± 0.07)
Pera glabrata	47.4 (± 0.43)	1.8 (± 0.11)	0.8 (± 0.18)	498.0 (± 22.04)	94.7 (± 6.29)	56.6 (± 3.10)	26.5 (± 1.58)	2.35 (± 0.65)
Myrcia glabra	47.7 (± 0.18)	1.6 (± 0.07)	0.9 (± 0.02)	309.5 (± 4.09)	161.3 (± 4.61)	100.5 (± 7.20)	29.4 (± 1.16)	1.85 (± 0.11)
Myrcia racemosa	41.6 (± 0.37)	1.4 (± 0.04)	0.5 (± 0.07)	96.9 (± 8.74)	124.1 (± 6.19)	115.0 (± 6.30)	30.2 (± 0.71)	2.71 (± 0.33)
Andira anthelmia	48.2 (± 0.20)	3.1 (± 0.10)	1.4 (± 0.12)	338.1 (± 3.74)	43.1 (± 2.58)	37.0 (± 2.33)	15.5 (± 0.51)	2.27 (± 0.17)
Abarema brachystachya	47.2 (± 0.07)	3.1 (± 0.10)	1.1 (± 0.09)	238.8 (± 2.12)	21.7 (± 1.29)	14.7 (± 0.94)	15.1 (± 0.49)	2.80 (± 0.17)
Cupania oblongifolia	48.2 (± 0.75)	2.0 (± 0.10)	1.3 (± 0.26)	362.3 (± 5.59)	84.0 (± 10.77)	69.7 (± 8.46)	24.1 (± 1.35)	1.56 (± 0.30)
Miconia sp.	44.3 (± 0.57)	1.8 (± 0.06)	0.8 (± 0.06)	308.1 (± 3.51)	101.1 (± 6.59)	91.5 (± 6.72)	24.3 (± 0.95)	2.31 (± 0.14)
Inga edulis	46.3 (± 0.35)	3.3 (± 0.10)	1.9 (± 0.20)	350.7 (± 0.88)	59.4 (± 8.71)	41.6 (± 6.48)	13.8 (± 0.50)	1.80 (± 0.19)
Lacistema pubescens	45.1 (± 0.15)	2.0 (± 0.08)	1.2 (± 0.02)	256.1 (± 18.07)	77.2 (± 5.83)	61.5 (± 4.67)	22.5 (± 0.80)	1.71 (± 0.04)

Table S2. Total abundance of macroinvertebrate morphospecies collected inside the coarse-mesh bags (n = 5) per time, litter treatment (LT) and temperature.

		time					LT					Temperature	
Family	Morphosp.	16 days	32 days	48 days	64 days	80 days	LT1	LT2	LT3	LT4	LT5	Ambient	Warming
Psycodidae	psychod_2	24	30	24	11	13	12	28	44	13	5	63	39
Tubificidae	oligoch_1	109	296	82	137	93	152	142	206	134	83	339	378
Chironomidae	orthoclad	46	157	171	167	130	92	144	188	118	129	406	265
Psycodidae	psychod_5	5	0	0	1	0	0	0	0	5	1	1	5
Limoniidae	trentepohlia	20	26	38	51	42	53	31	47	18	28	91	86
Chironomidae	p_kaingang	12	14	20	13	21	8	25	37	3	7	33	47
Psychodidae	psychod_4	8	2	0	0	0	1	2	6	1	0	3	7
Ceratopogonidae	atrichop_2	5	52	19	4	3	19	13	28	9	14	61	22
Chironomidae	p_marcond	6	73	84	100	69	31	57	70	97	77	101	231
Culicidae	culex_1	4	9	11	3	0	3	10	6	7	1	18	9
Culicidae	anopheles	2	0	1	0	1	0	0	0	2	2	2	2
Scirtidae	scirtes	3	3	11	11	3	3	6	3	12	7	11	20
Psychodidae	psychod_1	5	11	4	2	0	1	12	3	1	5	8	14
Sciaridae	sciarid	8	1	1	0	0	0	0	0	3	7	9	1
Ephydridae	ephydrid	1	0	0	0	0	0	0	1	0	0	0	1
Culicidae	culex_2	2	0	1	0	0	0	3	0	0	0	1	2
Ceratopogonidae	atrichop_5	1	14	5	3	0	3	1	4	2	13	17	6
Culicidae	wyeomyia	0	1	0	2	0	1	1	0	0	1	3	0
Ceratopogonidae	atrichop_3	0	6	7	3	0	4	0	3	3	6	3	13
Anisopodidae	olbiogaster	0	1	0	1	2	1	1	1	1	0	2	2
Tubificidae	oligoch_2	0	5	2	9	0	3	0	0	5	8	0	16
Culicidae	culex_5	0	0	3	0	1	0	0	0	3	1	4	0
Limnocytheridae	elpidium	0	0	1	0	0	1	0	0	0	0	0	1
Ceratopogonidae	atrichop_1	0	0	0	1	0	0	0	0	1	0	0	1
